# Endoscopic submucosal dissection for the treatment of a large inflammatory fibroid polyp in the gastric antrum prolapsing into the duodenum: A case report

**DOI:** 10.1097/MD.0000000000037877

**Published:** 2024-04-19

**Authors:** Xiaoyun Wang, Ru Ma, Jizhou Ma, Na Tang, Rui Li, Xiaojin Ma

**Affiliations:** a Department of Gastroenterology, The First People’s Hospital of Shizuishan, Affiliated to Ningxia Medical University, Shizuishan, China; b Department of Pathology, The First People’s Hospital of Shizuishan, Affiliated to Ningxia Medical University, Shizuishan, China.

**Keywords:** endoscopic submucosal dissection, inflammatory fibroid polyp, pyloric obstruction

## Abstract

**Rationale:**

Inflammatory fibroid polyp (IFP), also known as Vanek tumor, is a rare, benign gastrointestinal lesion characterized by its inflammatory and fibroid histological features. IFP is often discovered incidentally during endoscopic examinations. It is exceedingly rare for an IFP to prolapse into the duodenum and results in incomplete obstruction of the pylorus.

**Patient concerns:**

A 64-year-old male patient was admitted to the hospital with recurrent episodes of melena over a 6-month period, along with complaints of dizziness and fatigue in the past 10 days.

**Diagnoses:**

Gastroscopy showed a giant polypoid mass on the posterior wall of the gastric antrum, prolapsing into the duodenum. Abdominal computer tomography (CT) confirmed the tumor protruding into the duodenum. Pathologic examination of the resected specimen confirmed the IFP diagnosis.

**Interventions:**

The giant tumor was completely and successfully excised using endoscopic submucosal dissection (ESD). After the surgery, the patient underwent acid suppression and fluid replenishment therapy.

**Outcomes:**

The patient responded well to ESD and was discharged in stable condition. As of the submission of the case report, there has been no recurrence of the tumor after a 5-month follow-up, and the patient is still under follow-up.

**Lessons:**

While IFPs have traditionally been managed surgically, ESD demonstrates promising treatment outcomes, avoiding the need for surgical distal gastrectomy, and emerges as a safe and effective treatment option.

## 1. Introduction

In 1949, Vanek first described the case of inflammatory fibroid polyps (IFPs) under the nomenclature “submucosal granulomas with eosinophilic infiltration of the gastrointestinal tract.”^[1]^ It was in 1953 that Helwig and Ranier first employed the term IFP, which subsequently gained widespread acceptance.^[2]^ A large IFP in the gastric antrum, also known as a “Vanek tumor,” is a rare entity.^[3]^ Prolapse of IFP into the duodenum causing incomplete pyloric obstruction is exceptionally uncommon. Herein, we report a male patient presented with anemia due to surface ulceration and bleeding of the tumor. The tumor was completely excised through endoscopic submucosal dissection (ESD). Postoperative pathology confirmed the diagnosis of IFP, and the patient showed good recovery.

## 2. Case presentation

**Figure 1.**
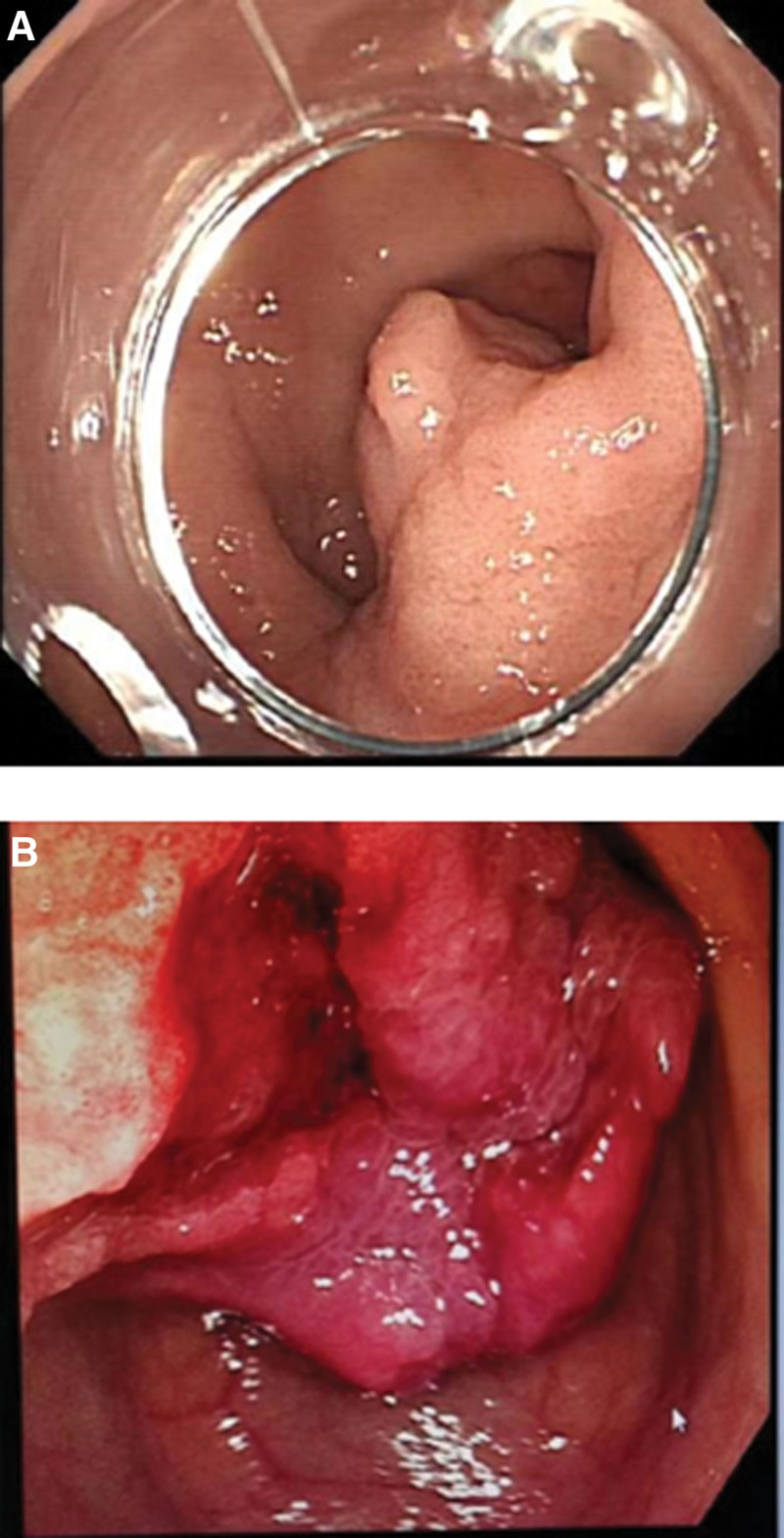
(A) Endoscopic view of the gastric cavity showing the lesion located in the gastric antrum. The surface mucosa is slightly edematous, with no clear boundaries of the lesion visible. (B) Endoscopic view of the duodenal bulb showing the lesion prolapsing from the pylorus into the duodenal bulb, with local surface ulceration.

**Figure 2.**
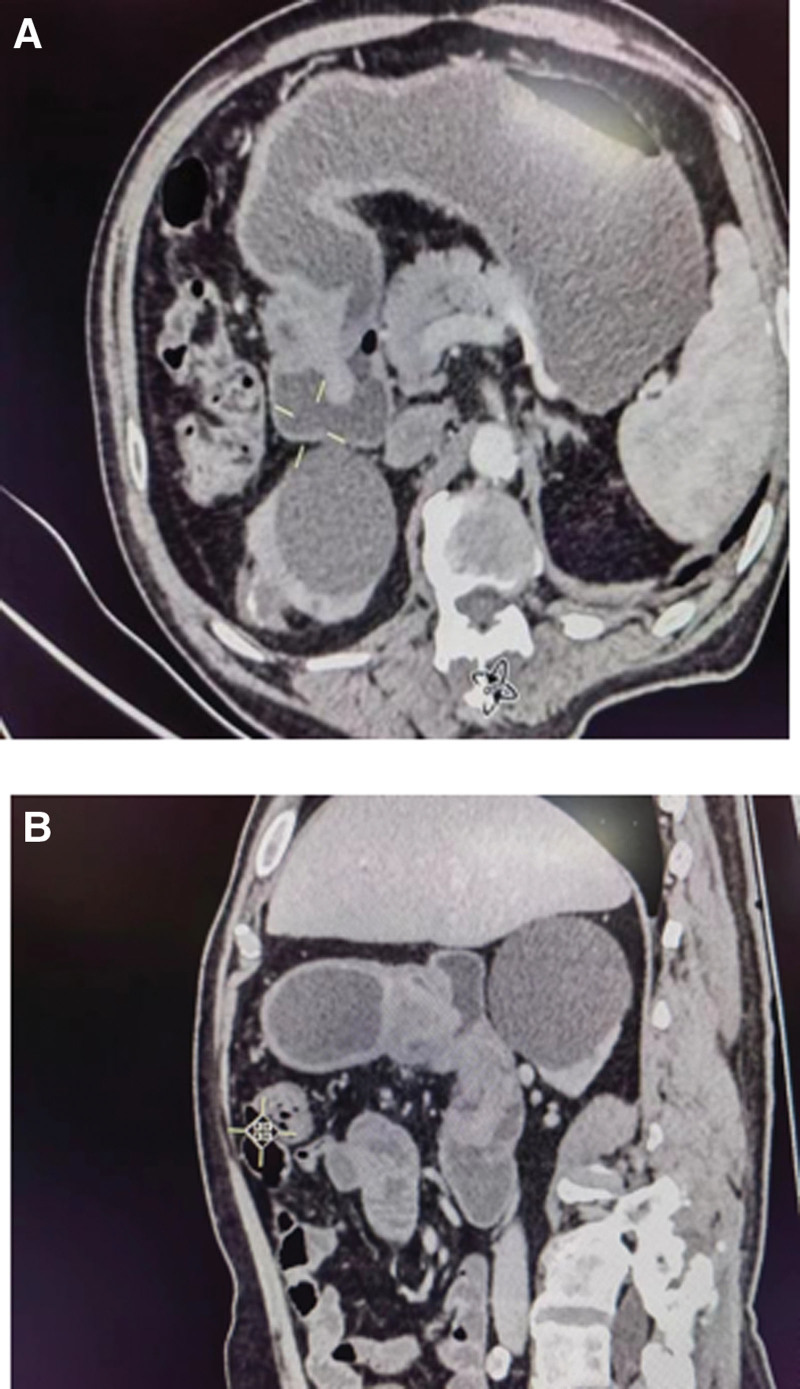
(A and B) Soft-tissue density shadow seen in the gastric pylorus and duodenum, connected to the base of the pyloric gastric wall, extending into the duodenum.

A 64-year-old male patient presented with recurrent episodes of melena for 6 months, accompanied by dizziness and fatigue for the past 10 days. Abdominal examination revealed a soft abdomen without tenderness, rebound pain, palpable masses, and normal bowel sounds. Laboratory tests showed a hemoglobin level of 5.6 g/L. C13 breath test for *Helicobacter pylori* was negative. Gastroscopy revealed a polypoid mass approximately 2 cm in diameter, located on the posterior wall of the gastric antrum on the greater curvature side, prolapsing into the duodenal bulb (Fig. 1A), causing incomplete pyloric obstruction with barely passable scope. The surface of the tumor was ulcerated with minor bleeding (Fig. 1B). Abdominal computer tomography (CT) scan showed the tumor prolapsing from the gastric antrum into the duodenal bulb and descending part (Fig. 2A and B). After a multidisciplinary team discussion, the lesion was scheduled. During the procedure, a transparent cap was utilized to peel off the tumor, thoroughly exposing the pylorus. The mucosa around the pyloric circumference at a 2 cm distance appeared normal. After adequate submucosal injection, an incision was first made on the side near the pylorus, followed by circumferential cutting of the mucosal edge of the tumor-wide base. The tumor was then peeled from the submucosa. During the peeling process, the tumor-feeding blood vessels were exposed and sufficiently coagulated before being severed. After peeling off nearly two-thirds of the lesion, the tumor was fully aspirated toward the gastric antrum side for complete removal. The procedure was carried out smoothly without significant bleeding. The specimen was retrieved orally using a snare and then pulled out of the body. The surgical site was adequately cauterized for hemostasis. Postoperatively, the patient was treated with acid suppression and fluid replenishment therapy.

**Figure 3.**
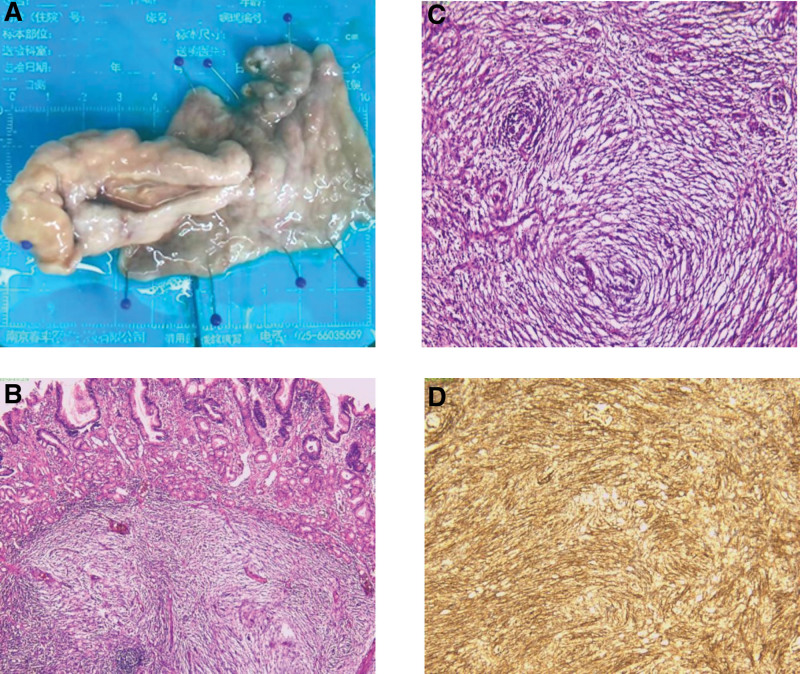
(A) Gross morphology of the lesion after fixation in formalin solution. (B) Microscopic view showing a well-demarcated lesion composed of spindle cells in the submucosa (magnification 10 × 4). (C) Microscopic view demonstrating the main cell types including fibroblasts, inflammatory cells, and proliferating blood vessels. Fibroblasts are arranged in a whorled pattern around blood vessels, forming concentric circles (magnification 10 × 30). (D) Immunohistochemical staining showing diffuse positive expression of CD34 (magnification 10 × 10).

Pathological examination revealed that the resected specimen grossly measured 8.6 cm × 6.5 cm, with the tumor measuring 7.3 cm × 3.3 cm × 2 cm (Fig. 3A). Microscopic evaluation of the submucosa displayed diffuse growth of spindle cells with infiltration of inflammatory cells and neovascularization. Fibroblasts showed whirlpool-like and concentric arrangements (Fig. 3B and C). Immunohistochemical analysis showed positivity for vimentin, CD34 (Fig. 3D), and smooth muscle actin, with a Ki67 index of 10% and positive staining for FVIII (vascular+). However, staining for desmin, CD117, DOG-1, and S-100 were negative. Pathological diagnosis: IFP of the gastric antrum.

The patient’s response to the endoscopic resection was favorable, leading to a stable discharge from the hospital. As of December 2023, the patient has been followed up for 4 to 5 months. At the 2-month follow-up, he did not report any discomfort. Repeat gastroscopy of the gastric antrum showed scar changes after ESD, with no local recurrence observed. The patient maintains a positive health status and the follow-up is still ongoing.

## 3. Discussion

The etiology of IFP remains unclear, but it may be associated with chemical, physical, metabolic factors,^[4]^ or *H. pylori* infection.^[5,6]^ Previously considered a benign proliferative disease, recent molecular biology research indicates that IFPs often harbor mutations in the platelet-derived growth factor receptor alpha gene, suggesting that they are neoplastic lesions caused by these mutations. Consequently, the 2010 fourth edition of the World Health Organization classification of digestive system tumors categorizes IFPs as benign tumors of the gastrointestinal tract.^[7]^ IFPs are predominantly seen in adults and are mostly asymptomatic, or they may cause abdominal pain due to gastric outlet obstruction, often incidentally discovered during endoscopic examinations.^[8]^

Grossly, IFPs appear as sessile, firm, gray-brown polypoid lesions or lesions with an incomplete stalk, which can be solitary or multiple. Most lesions occur in the gastric antrum, varying in size from <1 cm to 12 cm. IFPs originate from the submucosal layer, usually covered by normal mucosa, and resemble leiomyomas or gastrointestinal stromal tumors. Approximately one-quarter of cases show mucosal erosion. These lesions can extend to the muscularis propria and even to the serosa. Histologically, IFPs are composed of loosely structured connective tissue. The predominant cell types are spindle-shaped fibroblast-like cells, mixed with inflammatory cells and proliferating blood vessels, distributed within edematous stroma. Fibroblasts or myofibroblasts are arranged in a whorled pattern around thin-walled blood vessels, forming concentric circles or onion-skin patterns.^[9]^ The proliferating cells are uniform, with abundant cytoplasm and pale staining spindle-shaped nuclei. The tissue may be infiltrated with varying numbers of eosinophils and lymphocytes. The differential diagnosis of IFP includes eosinophilic gastroenteritis, gastrointestinal stromal tumors, and other mesenchymal lesions. Spindle cells in IFP show diffuse positivity for vimentin and CD34 immunoreactions. Histiocyte markers may show focal positivity. Alpha-smooth muscle actin may exhibit focal positivity. Cytokeratin, desmin, S-100, factor VIII, and Ki67 typically show negative reactions in spindle cells.^[10,11]^

Currently, surgical or endoscopic resection is the primary treatment method for IFP. In the case we reported, the tumor was large and prolapsed from the pyloric orifice, causing an incomplete pyloric obstruction. The mucosa on the gastric antrum side of the tumor was significantly edematous without obvious rupture, while on the side that prolapsed into the duodenum, the surface was ulcerated. Based on the CT findings, it was considered that the large size of the tumor led to the prolapse of the gastric antrum mucosa into the duodenal bulb, causing the tumor to cantilever. Prolonged cantilevering led to the shedding and necrosis of the surface mucosa, resulting in the patient’s melena. As there was no evidence of malignancy preoperatively and CT supported the presentation of gastric antrum mucosal prolapse, ESD was performed after a multidisciplinary team discussion. Intraoperatively, endoscopic hemostasis was achieved using electrocoagulation and hot biopsy forceps, and no complications such as perforation or bleeding occurred postoperatively. Although ESD for this giant IFP case shows good results, open surgery may provide a clearer view. For such cases, whether ESD or surgery is better requires prospective comparative studies to draw a reliable conclusion.

## 4. Conclusion

This case is a rare large IPF in the gastric antrum prolapsing into the duodenum, and the ESD treatment was effective, avoiding the need for distal gastrectomy. It serves as a valuable treatment option.

## Acknowledgments

We thank Dr Bin Qiao for editing this manuscript.

## Author contributions

**Conceptualization:** Jizhou Ma, Rui Li.

**Formal analysis:** Xiaoyun Wang, Jizhou Ma.

**Funding acquisition:** Na Tang.

**Investigation:** Jizhou Ma, Na Tang, Rui Li, Xiaojin Ma.

**Methodology:** Xiaoyun Wang, Na Tang.

**Resources:** Xiaojin Ma.

**Validation:** Ru Ma, Xiaojin Ma.

**Visualization:** Ru Ma.

**Writing—original draft:** Xiaoyun Wang, Ru Ma.
